# Comparison of effects of modafinil and caffeine on fatigue-vulnerable and fatigue-resistant aircrew after a limited period of sleep deprivation

**DOI:** 10.3389/fphys.2023.1303758

**Published:** 2024-01-08

**Authors:** Yara Q. Wingelaar-Jagt, Thijs T. Wingelaar, Wim J. Riedel, Johannes G. Ramaekers

**Affiliations:** ^1^ Center for Man in Aviation, Royal Netherlands Air Force, Soesterberg, Netherlands; ^2^ Department of Neuropsychology and Psychopharmacology, Faculty of Psychology and Neuroscience, Maastricht University, Maastricht, Netherlands; ^3^ Diving Medical Center, Royal Netherlands Navy, Den Helder, Netherlands

**Keywords:** aviation, fatigue, shift work, sleep, wakefulness-promoting agents, fatigue tolerance

## Abstract

**Introduction:** Literature suggests pilots experience fatigue differently. So-called fatigue-resistant or -vulnerable individuals might also respond differently to countermeasures or stimulants. This study, which is part of a larger randomized controlled clinical trial, aims to investigate the effect of caffeine and modafinil on fatigue-resistant and -vulnerable pilots.

**Methods:** This study included 32 healthy employees of the Royal Netherlands Air Force, who completed three test days, separated by at least 7 days. After a regular work day, the subjects were randomly administered either 300 mg caffeine, 200 mg modafinil or placebo at midnight. Hereafter the subjects performed the psychomotor vigilance test (PVT), vigilance and tracking test (VigTrack) and Stanford sleepiness scale (SSS) six times until 8 a.m. the next day. Subjects were ranked on the average number of lapses on the PVT during the placebo night and divided into three groups: fatigue-vulnerable (F_VUL_), -intermediate (FINT) and -resistant (F_RES_), with 11, 10 and 11 subjects in each group, respectively. Area under the curve (AUC) of the PVT, VigTrack and SSS during the test nights were calculated, which were used in univariate factorial analysis of variance (ANOVA). Tukey’s HSD *post hoc* tests were used to differentiate between the groups.

**Results:** A significant effect of treatment was found in the ANOVA of both PVT parameters, VigTrack mean reaction time and SSS. There was a statistically significant effect of fatigue group on all PVT parameters and VigTrack mean percentage omissions, where F_INT_ and F_RES_ scored better than F_VUL_. There was a significant interaction effect between treatment and fatigue group for PVT number of lapses. This is congruent for the AUC analyses in which for all parameters (except for the SSS) the performance of the F_VUL_ group was consistently worse than that of the F_INT_ and F_RES_ groups.

**Discussion:** This study demonstrates that the performance of individuals with different fatigue tolerances are differently affected by simulants after a limited period of sleep deprivation. The classification of fatigue tolerance through PVT lapses when sleep deprived seems to be able to predict this.

## 1 Introduction

With air travel in 2022 increasing to pre-COVID levels, the filing of fatigue reports has increased. In March 2022, Southwest Airline pilots filed 35 reports for every 10,000 duty periods, compared with 10 reports for every 10,000 duty periods in March 2019 (Wallace, 2022). Unfortunately, pilot fatigue not only leads to safety reports, but can and does result in incidents (Wingelaar-Jagt et al., 2021). A recent example is the Ethiopian Airlines pilots who fell asleep during flight and overflew their destination (Bekele, 2022). Pilots report that the currently high rate of experienced fatigue is most likely due to a shortage of pilots, combined with scheduling difficulties, which are issues that occur worldwide and are difficult to solve (Polek, 2022; Ziemelis, 2023). Even when duty time limitations are adhered to, these issues increase the workload of pilots, one of the contributing factors to fatigue as described by the International Civil Aviation Organization: *“A physiological state of reduced mental or physical performance capability resulting from sleep loss, extended wakefulness, circadian phase, and/or workload (mental and/or physical activity) that can impair a person’s alertness and ability to perform safety related operational duties”* (International Civil Aviation Organization ICAO, 2020).

However, not all pilots react the same to the fatigue; the level of experienced fatigue and subsequent effect on performance differs highly between individuals (Wingelaar-Jagt et al., 2021). These inter-individual differences in the level of fatigue experienced and the performance decrements are also found in (military) pilots, even in highly trained and selected individuals such as fighter pilots (Petrie et al., 2004; Van Dongen et al., 2006). These differences are robust and stable, i.e., they are most probably individual traits instead of consequences of analyses, sleep history or reactions to the type of sleep loss and therefore difficult to control or change (Van Dongen et al., 2004; Chua et al., 2019; Yamazaki and Goel, 2020). This level of vulnerability has been described as fatigue resistance and fatigue susceptibility, or fatigue-resistant and -susceptible/vulnerable individuals (Harrison et al., 2008).

Recent studies demonstrate that whether individuals are fatigue-resistant or -vulnerable might affect the rate with which stimulants like modafinil influences one’s performance under sleep deprivation (Caldwell et al., 2020; Van Cutsem et al., 2021). Stimulants are used to enhance the performance of fatigued (military) pilots, thereby mitigating the risks associated with fatigue (Institute of Medicine US Committee on Military Nutrition Research, 2001). If the effectiveness of stimulants depends on an individual’s fatigue susceptibility, determining if a pilot is fatigue-resistant or -vulnerable might be useful when advising the pilot whether to take a stimulant.

Unfortunately, distinct characteristics such as sex, race, age, and body mass index are not able to predict whether individuals are fatigue-resistant or -vulnerable or one’s response to stimulants (Yamazaki and Goel, 2020; Galli et al., 2022). Several methods have been introduced to identify fatigue-vulnerable and -resistant individuals (Yamazaki et al., 2022). A commonly used approach is to look at performance under sleep deprivation and rate individuals with good scores as fatigue-resistant and those with low scores as fatigue-vulnerable (Patanaik et al., 2015; Chua et al., 2019; Caldwell et al., 2020). Another method is to look at the change in performance under sleep deprivation compared with baseline, whereby individuals with little change are considered resilient (Patanaik et al., 2014; Riontino and Cavallero, 2021). A third suggested method is to look at intra-individual variance in performance (Yamazaki et al., 2022). Recent research indicates that all three approaches are comparable for psychomotor vigilance test (PVT) lapses; however, only the first method seems to be effective for subjective sleepiness metrics (Casale et al., 2022; Yamazaki et al., 2022). Predicting fatigue susceptibility by looking at baseline parameters yields promising results, but is not yet perfect (Patanaik et al., 2015; Galli et al., 2022).

This study is part of a larger randomized controlled trial that was designed to investigate several aspects of the implementation of modafinil and caffeine as countermeasures for fatigue in (military) aviation. In a previously published manuscript about this trial, we concluded that both modafinil and caffeine significantly decrease the negative effects of an extended period of continuous wakefulness on vigilance compared with a placebo (Wingelaar-Jagt et al., 2023). The present study compared the effects of modafinil and caffeine administration on the fatigue-vulnerable and -resistant participants of our population. We expected modafinil and caffeine to have a greater effect on the performance of fatigue-vulnerable individuals.

## 2 Materials and methods

This study is part of a larger randomized controlled trial, for an elaborate description of the materials and methods we therefore also refer to Wingelaar-Jagt et al. (2023) (Wingelaar-Jagt et al., 2023).

### 2.1 Participants

The larger randomized controlled trial conducted at the Center for Man in Aviation, Royal Netherlands Airforce (RNLAF; Soesterberg, the Netherlands) was carried out in accordance with the principles outlined in the Declaration of Helsinki. The study received ethical approval from the Medical Ethical Committee Brabant (reference: NL62145.028.17/P1749) and the Surgeon General of the Ministry of Defense (reference: DGO100117022). Informed consent was obtained from each participant. The trial was registered in the Dutch Trial Register (No. NTR6922) and EU Clinical Trials Register (No. 2017-002288-16).

Participation in this study was open to employees of the RNLAF, aged between 18 and 60 years, who met the aeromedical fitness criteria of the RNLAF Military Aviation Regulations or European Aviation Regulations (European Aviation Safety Authority EASA, 2011; Military Aviation Authority, 2020). Exclusion criteria primarily revolved around potential interactions or side effects involving caffeine or placebo, including conditions such as pregnancy or breastfeeding, usage of medications metabolized by CYP3A4/5, CYP2C19, or CYP2C9 enzymes, and psychiatric disorders (e.g., sleep disturbances).

Upon receiving comprehensive verbal and written information detailing the objectives, implications, and limitations of the trial, all participants provided written consent. This consent was given on a voluntary basis and could be withdrawn at any point without any adverse consequences. In adherence to both national and international privacy regulations, no study-related data were incorporated into the participants’ medical records.

The trial included 32 subjects: two subjects did not participate in the caffeine condition due to operational reasons. According to the design protocol of the study, their test results were included in the analysis. The subjects characteristics are equal to those described in the article about the comparison between the effects of modafinil and caffeine with placebo on night-time vigilance (Wingelaar-Jagt et al., 2023): Subjects’ ages ranged from 25 to 59 years (median age: 30.9 years, IQR: 28.9–39.3 years). Among the 32 subjects, five (16%) were female, and a majority of 21 (66%) were pilots. On trial days, the median waking time of the subjects was 07:00 a.m. (IQR: 06:00–07:30 a.m.), meaning that at T0 the subjects had a median period of wakefulness of 17 h (IQR: 16.5–18.0 h).

### 2.2 Study drugs

Caffeine is a widely accepted, available, and well-known stimulant (McLellan et al., 2016). It is a nonprescription stimulant that blocks adenosine receptors (Daubner et al., 2021). Absorption of caffeine (usually in the range of 200–600 mg) is rapid (15–40 min), and its effects are observable 15–20 min after administration (Caldwell et al., 2009). With a half-life of 4–6 h, it improves vigilance until approximately 8 h after administration (Klopping et al., 2005).

Modafinil, usually at a dose of 100–200 mg, is a newer stimulant that is already used as a fatigue countermeasure in the air forces of Singapore, the United States, India, and France (Ooi et al., 2019). It is a prescription drug, that in the United States is FDA-approved for the treatment of narcolepsy, sleep work shift disorder and obstructive sleep apnea in adults. Although its exact biochemical process is unknown, it is thought to alter the height of different neurotransmitters (e.g., serotonin, noradrenalin, dopamine, and gamma-aminobutyric acid (Kim, 2012; Battleday and Brem, 2015). Its effectiveness as a countermeasure has been demonstrated in several studies with different periods of wakefulness (Wesensten et al., 2004; Estrada et al., 2012; Wingelaar-Jagt et al., 2023).

Capsules that only contained a filler and no active substance were used as the placebo for comparison.

### 2.3 Materials

On the trial days, several parameters were measured six times: baseline measurement at 6 h (T-6) before administering trial medication (T0) and at 1, 2, 3, 4, 6 and 8 h after T0 (T1, T2, T3, T4, T6 and T8, respectively).

The Vigilance and Tracking test (VigTrack) is a dual-task that measures vigilance performance under the continuous load of a compensatory tracking task. The test has been used in various studies and is sensitive for measuring vigilance and alertness (Valk and Simons, 2009; Simons, 2017). During the tracking task, participants had to steer a blue dot using a joystick such that it remained below a red dot in the center of the display. The blue dot is programmed to move continuously from the center of the display. While tracking, participants had to perform an additional vigilance task. Inside the red dot, a black square alternated with a diamond, once per second. At random intervals, a hexagon was presented. When this occurred, participants had to press an additional key on the joystick. The duration of this test was 10 min, and primary endpoints included root mean square tracking error, percentage omissions and mean reaction time. At the start of every trial day, three familiarization sessions of 5 min were scheduled for all subjects to avoid practice bias during the actual measurements.

The PVT measures the speed with which subjects respond to a red stimulus and is used to assess the vigilance of subjects (Basner and Dinges, 2011). The inter-stimulus interval, defined as the period between the last response and the appearance of the next stimulus, varies randomly from 2 to 10 s. The duration of this test was 10 min, and primary endpoints included reaction time and lapses. Lapses (errors of omission) were defined as reaction times ≥500 msec. At the start of every trial day, a familiarization session of 5 min was scheduled for all subjects to avoid practice bias during the actual measurements.

The Stanford Sleepiness Scale (SSS) was used to subjectively assess the degree of sleepiness in subjects during the test days (Hoddes et al., 1973). This subjective rating scale is sensitive to detect any significant increase in sleepiness or fatigue, and is highly correlated with flying performance and the threshold of information-processing speed during periods of intense fatigue (Perelli, 1980).

### 2.4 Design

The randomized controlled trial encompassed a series of three nonconsecutive trial days for each subject, during which capsules of modafinil, caffeine, or placebo were administered once immediately after midnight. Details of the trial days can be found in Table 1 and a previously published article by Wingelaar-Jagt et al. (2023). The modafinil dosage administered was 200 mg, a recognized effective fatigue mitigation measure for military aviators (Caldwell et al., 2000; Caldwell et al., 2009). The dosage of caffeine (300 mg) corresponded to the standard dosage presently administered to RNLAF aviators, representing a medium-range yet efficacious amount (Lohi et al., 2007; Caldwell et al., 2009).

**TABLE 1:** Overview of study design and data collection. All study days were identical, the only difference being the medication administered.

Timing	Activity
The 3 days before every trial day	Sleep diary
	Caffeine log
4:30 p.m.	Vital parameters
	Stanford Sleepiness Scale
	Familiarization with PVT and VigTrack
5:00 p.m.	Subject ceased caffeine consumption
6:00 p.m.	**Baseline block (T-6)**
	Stanford Sleepiness scale
	Assessment of VigTrack and PVT
Midnight	**Second baseline block (T0)**
	Vital parameters
	Stanford Sleepiness scale
	Assessment of VigTrack and PVT
	Blood samples
	**Test medication administration**
1:00 a.m.	**First test block (T1)**
	Stanford Sleepiness scale
	Assessment of VigTrack and PVT
2:00 a.m.	**Second test block (T2)**
	Vital parameters
	Stanford Sleepiness scale
	Assessment of VigTrack and PVT
3:00 a.m.	**Third test block (T3)**
	Stanford Sleepiness scale
	Assessment of VigTrack and PVT
	Blood samples
4:00 a.m.	**Fourth test block (T4)**
	Stanford Sleepiness scale
	Assessment of VigTrack and PVT
6:00 a.m.	**Fifth test block (T6)**
	Stanford Sleepiness scale
	Assessment of VigTrack and PVT
	Blood samples
8:00 a.m.	**Sixth test block (T8)**
	Vital parameters
	Stanford Sleepiness scale
	Assessment of VigTrack and PVT
	Blood samples
Outtake	Sleep questionnaires

A wash-out interval of no less than 7 days, as advised by our pharmacist, was instituted to ensure complete drug elimination and to prevent any interference with subsequent trial day analyses. The trial was double-blinded to ensure that both participants and investigators remained uninformed about the treatment assigned on trial days. The sequence of treatments (placebo, caffeine, or modafinil) for each individual was determined through a computer-generated randomization schedule, organized and overseen by an external statistician. This randomization encompassed all feasible (six) treatment sequences, thus promoting equilibrium in terms of carryover effects; factors like skill enhancement or learning bias on the test battery. In preparation for each trial day, researchers obtained a treatment kit from the pharmacist, featuring capsules that were identical and labeled with the respective subject number and trial day.

### 2.5 Procedure

In the week leading up to each trial day, participants adhered to the local time zone of the research center (daylight saving GMT +2) to preemptively counter the potential influence of jetlag, which could introduce confounding variables to the test outcomes. Throughout the trial days, participants were instructed to refrain from engaging in strenuous physical activities, including sports, and from sleeping. They diligently maintained a record of their activities and documented their caffeine consumption. Regular consumption of their habitual amount of caffeine-containing products was permitted up until 5:00 p.m. To prevent caffeine from affecting vigilance, participants refrained from further caffeine intake after 5:00 p.m. on trial days.

Vital parameters such as temperature, blood pressure, and pulse were assessed on four occasions during each trial day: twice before the administration of medication, and at two and eight h post-administration (refer to Table 1 for details). In addition, female subjects underwent pregnancy tests on each trial day, and all participants were queried about any recent usage of concomitant medications or any unauthorized substances within the preceding 3 days. Participants were inquired about any adverse events multiple times during the course of the trial days and at each visit post-screening. Any adverse events that occurred during the study were recorded.

### 2.6 Statistical analysis

Differences of baseline characteristics (age, sex, function, waking time and wakefulness time) were tested using the Student’s t-test or Kruskal–Wallis test where appropriate.

Responses in the aforementioned tests (VigTrack, PVT and SSS) were collected during the night. An area under the curve (AUC) based on the results during the test night was calculated for each of these parameters, using the delta score. The delta score is corrected for the baseline scores at T-6 of each test subject (thus; Delta score = score at the respective test moment–the score of that individual at T-6).

Statistical analyses were performed using IBM SPSS Statistics for Windows, version 27.0 (Armonk, NY: IBM Corp, 2020). A univariate factorial analysis of variance (ANOVA) using the AUC was conducted to analyze the effects of group (F_VUL_, F_INT_, and F_RES_) and treatment (modafinil, caffeine, and placebo), and the interaction thereof. When the ANOVA revealed a significant effect, Tukey’s HSD *post hoc* test was utilized to analyze the difference between the different treatments or groups. A *p*-value <0.05 was considered statistically significant.

## 3 Results

The administration of the drugs did not exert any discernible impact on the subjects’ vital parameters. Throughout the trial, no adverse events were reported. The trial concluded in alignment with the protocol.

### 3.1 Characterization of fatigue resistant and fatigue vulnerable individuals

On the placebo test night, 224 PVT tests were performed by all subjects from T0 to T8. The Kolmogorov-Smirnov and Shapiro-Wilk tests of these data yielded *p*-values <0.001, indicating that these data were non-normally distributed. The median number of lapses was 13 (IQR: 6–24, range: 0–60).

To define participants as fatigue-resistant or -vulnerable, the average numbers of PVT lapses scored at T0 until T8 (h 17–25 of the sleep deprivation) during the placebo night were ranked. The fatigue-vulnerable (F_VUL_) group was defined as the lowest scoring third of participants, the fatigue-resistant (F_RES_) group as the highest scoring third, and the fatigue-intermediate (F_INT_) group as participants in between. In the case of ties, participants were placed in the better scoring group. This classification gave the following number of participants in each group: F_VUL_, n = 11; F_INT_, n = 10; and F_RES_, n = 11.

Of the 11 fatigue-resistant participants, one missed the caffeine administration, similar to the F_VUL_ group. Median age was higher in the F_VUL_ group than in the F_RES_ group, and fatigue-vulnerable participants had a slightly longer period of wakefulness at T0. The baseline characteristics of the three groups are shown in Table 2. Sex, age, and waking time (and derived period of wakefulness) did not significantly differ between the F_VUL_, F_INT_, and F_RES_ groups according to the Kruskal–Wallis test, with *p*-values of 0.329, 0.194, and 0.647, respectively.

**TABLE 2:** Comparison of characteristics of the three fatigue groups.

	Missed caffeine administration	Female	Median age	Median waking time	Median period of wakefulness at T0
**Total (n = 32)**	2 (6%)	5 (16%)	30.9 years (IQR: 29–39)	07:00 a.m. (IQR: 06:00–07:30)	17.0 h (IQR: 16.5–18.0 h)
**Fatigue-resistant (n = 11)**	1 (9%)	1 (9%)	31.4 years (IQR: 30–50)	07:20 a.m. (IQR: 06:12–07:30)	16.7 h (IQR: 16.5–17.8 h)
**Fatigue-intermediate (n = 10)**	0 (0%)	0 (0%)	29.2 years (IQR: 28–33)	06:57 a.m. (IQR: 06:00–07:30)	17.0 h (IQR: 16.5–18.0 h)
**Fatigue-vulnerable (n = 11)**	1 (9%)	4 (36%)	33.0 years (IQR: 30–36)	06:35 a.m. (IQR: 06:15–07:00)	17.4 h (IQR: 17.0–17.8 h)

### 3.2 Effect of fatigue group on treatment effects

**FIGURE 1:**
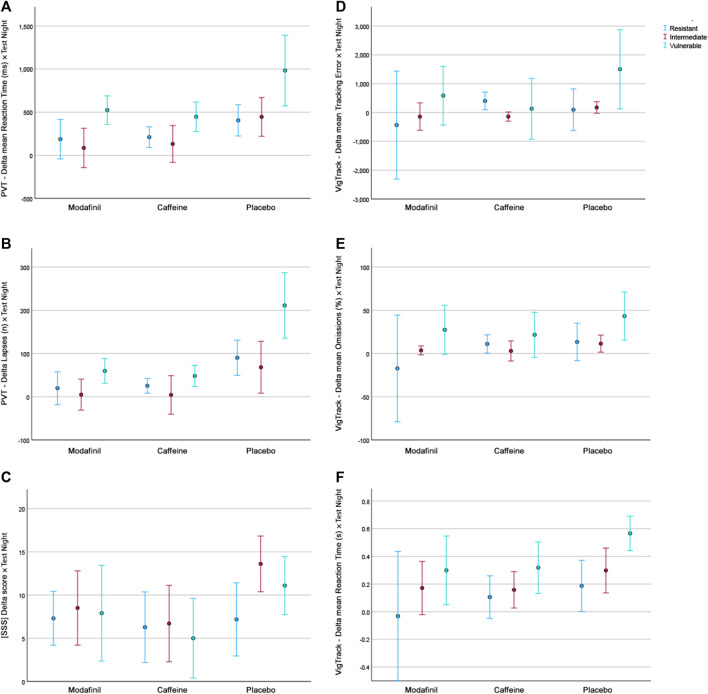
The mean AUC of outcome parameters according to treatment and fatigue group. **(A)**. PVT–Delta mean Reaction Time. **(B)**. PVT–Delta Lapses. **(C)**. Delta SSS scores. **(D)**. VigTrack–Delta mean Tracking Error. **(E)**. VigTrack–Delta mean Percentage Omissions. **(F)**. VigTrack–Delta mean Reaction Time. Blue = fatigue-resistant group, red = fatigue-intermediate group, green = fatigue-vulnerable group.

The mean AUC of outcome parameters according to treatment and fatigue group are shown in Figure 1. The results of univariate factorial ANOVAs and subsequent *post hoc* tests are displayed in Table 3.

**TABLE 3:** Outcomes of the Univariate factorial ANOVAs.

Covariates		PVT–mean reaction time	PVT–number of lapses	SSS	VigTrack–mean tracking error	VigTrack–mean percentage omissions	VigTrack–mean reaction time
Main effect of treatment		F (2, 9) = 11.448, *p* < 0.001^a^	F (2, 9) = 24.101, *p* < 0.001^a^	F (2, 9) = 4.829, *p* = 0.010^a^	F (2, 9) = 1.546, *p* = 0.219	F (2, 9) = 1.680, *p* = 0.192	F (2, 9) = 3.464, *p* < 0.036^a^
**Pairwise comparisons**	modafinil vs. placebo	*p* < 0.001^a^	*p* = 0.036^a^	*p* < 0.001^a^	n.a	n.a	*p* = 0.200
	caffeine vs. placebo	*p* < 0.001^a^	*p* = 0.130	*p* < 0.001^a^	n.a	n.a	*p* = 0.009^a^
**Main effect of fatigue group**		F (2, 9) = 15.894, *p* < 0.001^a^	F (2, 9) = 13.680, *p* < 0.001^a^	F (2, 9) = 1.566, *p* = 0.215	F (2, 9) = 3.021, *p* = 0.054	F (2, 9) = 4.911, *p* = 0.010^a^	F (2, 9) = 7.444, *p* < 0.001^a^
**Pairwise comparisons**	vulnerable vs. intermediate	*p* < 0.001^a^	*p* = 0.060^a^	*p* < 0.001^a^	n.a	*p* = 0.038^a^	n.a
	vulnerable vs. resistant	*p* < 0.001^a^	*p* < 0.001^a^	*p* < 0.001^a^	n.a	*p* = 0.016^a^	n.a
	intermediate vs. resistant	*p* = 0.829	*p* = 0.317	*p* = 0.431	n.a	*p* = 0.953	n.a
**Interaction effect**		F (4, 9) = 0.797, *p* = 0.530	F (4, 9) = 0.317, *p* = 0.866	F (4, 9) = 2.528, *p* = 0.046^a^	F (4, 9) = 1.156, *p* = 0.336	F (4, 9) = 0.642, *p* = 0.634	F (4, 9) = 0.913, *p* = 0.460

^a^ Statistically significant results (*p < .05*) from the Univariate factorial ANOVA, or subsequent Tukey HSD, *post hoc* tests.

For most parameters, except for the VigTrack mean tracking error and mean percentage omissions, there was a significant main effect of treatment. For the majority of the significant results, the subsequent *post hoc* tests showed that outcomes were significantly better after modafinil and caffeine administration than after placebo administration.

There was no significant main effect of fatigue group for SSS or VigTrack mean tracking error; the main effect of fatigue group was significant for the other four parameters. Subsequent *post hoc* tests revealed that scores were significantly better in the F_INT_ and F_RES_ groups than in the F_VUL_ group. There were no significant differences between the F_INT_ and F_RES_ groups.

There was a significant interaction effect between treatment and fatigue group for PVT number of lapses. This indicates that for this parameter the treatment did have a significantly different effect depending on the fatigue group. This is congruent with Figure 1B, in which the performance of the F_VUL_ group seems to be steadily lower than that of the F_INT_ and F_RES_ groups. This was especially pronounced after placebo administration. Furthermore, the effect of modafinil and caffeine administration appeared to be more extensive on scores in the F_VUL_ group than in the F_INT_ and F_RES_ groups. These trends in Figure 1 are comparable for most parameters (except for the SSS). For the PVT mean reaction time and VigTrack parameters, the scores in the F_VUL_ group after modafinil or caffeine administration were even similar to (or worse than) those in the F_INT_ and F_RES_ groups after placebo administration. However, the univariate factorial ANOVAs showed no significant interaction effect between treatment and fatigue group for the other parameters.

## 4 Discussion

This study demonstrates that individuals with different fatigue tolerances react differently to the negative effects of a limited period of sleep deprivation on performance. The classification of fatigue tolerance according to PVT lapses when sleep-deprived seems to be able to predict this. In a previously published manuscript about this trial, we concluded that subjects administered modafinil or caffeine showed greater vigilance after an extended period of continuous wakefulness than those administered a placebo (Wingelaar-Jagt et al., 2023). The current study indicates that the extent to which stimulants improve performance might depend on the fatigue tolerance of the individual. Individuals with a low fatigue tolerance (i.e., fatigue-vulnerable individuals) seem to benefit more from stimulant administration, while individuals who are fatigue-resistant or -intermediate generally retain higher performance than the fatigue-vulnerable individuals, regardless of the intervention.

PVT lapses during sleep deprivation showed strong interindividual differences in our study, in line with the literature (Van Dongen et al., 2004; Chua et al., 2019). Congruent with previous studies, we split the participants into three groups based on the number of participants after ranking their performance (Chua et al., 2019; Caldwell et al., 2020; Galli et al., 2022). However, the number of groups in previous studies varied between two and four, resulting in different cut-off values for the groups. These different classifications of fatigue vulnerability make it difficult to compare the results. While the three fatigue groups were statistically comparable at baseline, the median period of wakefulness was slightly longer in the F_INT_ and F_VUL_ groups than in the F_RES_ group. This is similar to a finding of Caldwell et al., who found a non-statistically significant difference in hours slept in the three nights prior to the test. However, low-scoring performers had obtained more hours of sleep in their study (Caldwell et al., 2020).

In concordance with previous research, we found that fatigue tolerance classification through PVT lapses when sleep-deprived seems to be valid (Patanaik et al., 2015; Chua et al., 2019; Yamazaki et al., 2022). The fatigue group had a significant effect on the majority of the parameters, with the exception of SSS and VigTrack mean tracking error, with the latter *p*-value approaching 0.05. The current study showed a significant interaction effect between treatment and fatigue group for PVT number of lapses. This indicates that for this single parameter the treatment did have a significantly different effect depending on the fatigue group. This is congruent with Figure 1, in which for all parameters (except for the SSS) the performance of the F_VUL_ group was consistently worse than that of the F_INT_ and F_RES_ groups. However, the ANOVAs showed no significant interaction effect between treatment and fatigue group for the other parameters. This discrepancy between the findings of the univariate factorial ANOVAs and visual depiction of the mean AUC of the different parameters according to the treatment and fatigue group may be because the direction of the effect of treatment is similar for the three fatigue groups. As Figure 1 indicates that the effect of modafinil and caffeine administration on scores appeared to be more extensive in the F_VUL_ group than in the F_INT_ and F_RES_ groups (except for the SSS), it might solely be the size of the effect that is different. Naturally, as the F_VUL_ group had a lower performance after placebo administration, there is more room in this group for performance improvements due to stimulant administration than in the F_INT_ and F_RES_ groups. This discrepancy is not as pronounced in our study as it was in that of Caldwell et al., who reported that high-performing individuals did not benefit substantially from modafinil administration while low-performing individuals did (Caldwell et al., 2020). However, for the PVT mean reaction time and VigTrack parameters, scores in the F_VUL_ group after modafinil or caffeine administration were similar to (or worse than) those in the F_INT_ and F_RES_ groups after placebo administration. This suggests that even though modafinil and caffeine improve performance, regardless of fatigue tolerance, performance of Fvul individuals remain lower after stimulant administration than that of Fres individuals without stimulants. This raises the question whether fatigue tolerance should be part of the selection process for individuals who regularly have to perform while fatigued (like military pilots on deployment).

Naturally, this study has some limitations. First, there is possible selection bias; the subjects were all military, aeromedically screened, and predominantly young. This is the population of interest for the Royal Netherlands Air Force, but this makes the results difficult to extrapolate to the general population. Furthermore, even though there was high motivation among our population to participate in this study, individuals who are uncomfortable with staying awake an entire night (possibly because they are fatigue-vulnerable) might be less inclined to participate in a sleep deprivation study. Second, the lack of a standard method and classification of fatigue groups makes it difficult to compare this research with previous studies. It would be favorable to introduce a classification including cut-off values that can be used in future studies to identify fatigue-resistant and -vulnerable individuals in order to increase comparability. Third, this study was performed in a controlled laboratory environment and used relatively simple tasks such as the PVT and VigTrack. Although both tests are sensitive for measuring vigilance and alertness, the results cannot be simply extrapolated to real-life scenarios because the workload and complexity of tasks in the cockpit are of a different caliber, which might influence individuals’ reactions to fatigue (Caldwell and Roberts, 2000; Ehlert and Wilson, 2021). Lastly, this study induced a rather limited duration of total sleep deprivation. Although research suggests that fatigue tolerance is consistent across different types of sleep deprivation, these findings might not accurately predict the response to other types of sleep deprivation, like chronic sleep restriction.

In conclusion, this study shows that fatigue tolerance classification through PVT lapses when sleep-deprived seems to predict the performance of other psychometric parameters of individuals when sleep-deprived. The importance of fatigue and its negative effects on performance is not limited to (military) aviation. In industries such as healthcare and logistics, in which peak performance is required during night-time or after periods of sleep deprivation, it is equally important to be able to identify which individuals might be at risk of performance decrements. To harmonize research into fatigue vulnerability, the identification of fatigue tolerance groups must be standardized and the introduction of a classification including cut-off values is paramount. The present study confirms that individuals have different degrees of performance degradation during a limited period of sleep deprivation and that, depending on the fatigue tolerance of the subject, stimulants might correct this to different extents. Stimulants might be especially useful for fatigue-vulnerable individuals, even though their performance after stimulant administration may remain lower than that of fatigue-resistant individuals when sleep-deprived.

## Data Availability

The datasets presented in this article are not readily available because of policy and privacy restrictions. Requests to access the datasets should be directed to the corresponding authors.
